# Audio-vestibular and radiological analysis in Meniere’s disease

**DOI:** 10.1016/j.bjorl.2022.08.003

**Published:** 2022-10-11

**Authors:** Arzu Kirbac, Saziye Armagan Incesulu, Ugur Toprak, Hamdı Caklı, Hulya Ozen, Suzan Saylisoy

**Affiliations:** a Eskişehir Osmangazi University, Faculty of Health Sciences, Department of Audiology, Eskişehir, Turkey; b Eskisehir Osmangazi University, Faculty of Medicine, Department of Otolaryngology, Eskişehir, Turkey; c Eskisehir Osmangazi University, Faculty of Medicine, Department of Radıology, Eskişehir, Turkey; d University of Health Sciences, Gulhane Faculty of Medicine, Department of Medical Informatics, Ankara, Turkey

**Keywords:** Meniere’s disease, Intravenous gadolinium-enhanced inner ear magnetic resonance imaging, Endolymphatic hydrops, Perilymphatic enhancement, Vestibular tests

## Abstract

**Objective:**

Intravenous gadolinium-enhanced inner ear magnetic resonance imaging (IV Gd-enhanced inner ear MRI) is a new technique for diagnosing Meniere’s disease (MD). Vestibular tests have also long been used for MD, but which tests should be included in the oto-neurological test battery remains controversial. The evaluation method to be used to confirm the clinical diagnosis in MD is not clear. This study aimed to examine the results of vestibular tests and IV Gd-enhanced inner ear MRI in individuals diagnosed with unilateral definite Meniere's disease.

**Methods:**

IV Gd-enhanced inner ear MRI (Endolymphatic Hydrops [EH] and Perilymphatic Enhancement [PE]), conventional audiometry (0.25–8 kHz), video Head Impulse Test (vHIT), cervical Vestibular Evoked Myogenic Potential (cVEMP), air caloric test, and dizziness handicap inventory were applied to 16 adult patients diagnosed with unilateral definite MD.

**Results:**

Among the patients with definite MD, EH (cochlear and/or vestibular) was identified in 93.7% and 68.7% of the symptomatic and the asymptomatic ears, respectively. There was a positive correlation between the hearing thresholds at 2, 4, 6 and 8 kHz and the degree of cochlear EH (*p* < 0.05). PE (cochlear and/or vestibular) was observed in 37.5% of the asymptomatic and symptomatic ears. The sensitivity of the vestibular test battery (vHIT, cVEMP, and caloric test) was 100% and its specificity was 50%, while the sensitivity of the IV Gd-enhanced inner ear MRI (EH and PE together) was 93.8% and the specificity was 81.3%.

**Conclusion:**

MRI had higher sensitivity and specificity than the vestibular test battery. PE or vHIT alone was not considered to be reliable in the diagnosis of MD. In suspected MD, the clinical history, hearing tests, and IV Gd-enhanced inner ear MRI are sufficient for diagnosis. If MRI technique is not possible, vestibular tests (caloric test and cVEMP, not vHIT) can provide reliable results when evaluated together.

## Highlights

• IV Gd-enhanced inner ear MRI (EH + PE) had higher sensitivity and specificity than the vestibular test battery.
• Among the vestibular tests, the highest sensitivity and specificity were obtained from the caloric test.
• In suspected MD, the clinical history, hearing tests, MRI (EH+PE) are sufficient for diagnosis in certain patient groups.

## Introduction

Meniere’s disease (MD) is a chronic disease.^1^ Endolymphatic Hydrops (EH) is accepted as the pathological hallmark in MD. In routine clinical practice, the principal role of temporal Magnetic Resonance Imaging (MRI) is to exclude other pathologies mimicking MD.^2^ Recently, the use of temporal MRI for the visualization of EH is under discussion.^2^, ^3^ Although postmortem histopathological studies have proven the presence of EH in the cochlear canal and vestibular organs at the symptomatic side of patients with MD.^4^, ^5^ Intravenous (IV) Gadolinium (Gd)-enhanced inner ear MRI studies have reported EH percentages varying in a broad range in both symptomatic and asymptomatic sides; therefore, there are conflicting data in the literature.^6^, ^7^

Four-hour delayed IV contrast MRI results in the deposition of diluted contrast in the perilymphatic space of the inner ear, outlining the impermeable endolymphatic compartment and allowing for the visualization of EH.^8^ Together with EH, recently, the inter-ear comparison of increased Perilymphatic Eenhancement (PE) (degree of accumulation of contrast in the perilymphatic area) has been suggested as a parameter to improve diagnostic accuracy of this technique in patients with suspected MD.^9^, ^10^, ^11^

In clinical practice, although history, symptoms, and audio-vestibular tests are utilized in diagnosis, and which tests should be included in the oto-neurological test battery remains controversial.^12^, ^13^ The diagnosis process has been further complicated by the addition of inner ear MRI with contrast enhancement, including various techniques.^14^ There are also limited studies examining both contrast-enhanced inner ear MRI and audio-vestibular tests in MD.^8^, ^14^, ^15^

In this study, it was aimed to examine the results of vestibular tests and IV Gd-enhanced inner ear MRI in individuals diagnosed with unilateral definite MD.

## Methods

### Subjects

Sixteen patients (mean age, 42.7 years) who were diagnosed with unilateral definite MD^1^ underwent IV Gd-enhanced inner ear MRI, audio-vestibular tests, and Dizziness Handicap Inventory (DHI). The side with increased (worse) hearing thresholds, tinnitus, and ear fullness was considered as the symptomatic ear, while the side with normal hearing thresholds and no ear fullness or tinnitus was accepted as the asymptomatic ear. The exclusion criteria were as follows: middle ear problems or presence of an air-bone gap in an audiogram, presence of a systemic or metabolic disease (such as diabetes mellitus and hypertension), use of an ototoxic drug (loop diuretic, etc.), history of an ear operation, and being under active medical and/or intratympanic treatment (diuretic, vestibulosuppresant, etc.). All assessments were made during the inactive period of MD (at least three weeks after the last vertigo attack). Written informed consent was obtained from all the participants, and approval was received from the local ethics committee.

### Inner ear MRI and image analysis

All MRI scans were performed using a 3T scanner (GE Discovery 750 w, General Electric, US) with a 32-channel head coil four hours after the administration of double-dose IV Gd (maximum perilymphatic contrasting time) (0.2 mmoL/kg from the contrast agent of the volume of 1.0 mmoL/mL). EH was evaluated by the visual comparison of the endolymphatic compartment through which the contrast agent could not pass and accumulated in the perilymphatic compartment in the cochlea and vestibule on the 3D FLAIR images obtained from the axial plane. Cochlear EH (cEH) was evaluated according to the criteria described by Barath et al. as no cEH, grade I, and grade II,^16^ and vestibular EH (vEH) was classified according to the modified Barath system defined by Bernaerts et al. as normal, grade I, grade II, and grade III.^2^ EH was considered to be present when either cEH or vEH or both were detected.^10^ The degree of PE was also evaluated semi-quantitatively by visually comparing the findings with the contralateral ear. The degree of PE was divided into three groups for both the vestibule and the cochlea: equal, less, and more. Since no perilymphatic gap is present in cases of third-degree vestibular hydrops, the evaluation of vestibular PE was accepted as Not-Applicable (N/A).^2^ EH and PE were evaluated by a radiologist with 20 years of experience, who was certified by the neuroradiology board and blinded to the clinical details of the patients.

### Audiological and vestibular tests

We determined the air-conduction hearing thresholds at 0.125 to 8 kHz, and the bone-conduction hearing thresholds at 0.5 to 4 kHz in both ears of all the participants. A clinical audiometer (AC 40 model, Interacoustics, Otometrics, Taastrup, Denmark) and supra-aural Telephonics TDH-39P earphones were used for pure-tone audiometry. Audiometry was performed by an audiologist. All the hearing thresholds were determined following a standard Hughson-Westlake procedure. Type A tympanograms were obtained with a 226-Hz probe tone (AZ26 clinical impedance audiometer; Interacoustics, Assens, Denmark), and middle ear function was accepted to be normal in cases where there was no air-bone gap in the audiogram. The Pure Tone Average (PTA) values of the low (0.25, 0.5, and 1 kHz), middle (0.5, 1, and 2 kHz) and high (2, 4, 6, and 8 kHz) air conduction thresholds were also calculated.

The video Head Impulse Test (vHIT) was used to test the function of each of the six SSCs.^17^ The Vestibulo-Ocular Reflex (VOR) gain of SSCs and the existence of saccades are examined using vHIT (ICS impulse system; GN Otometrics, Taastrup, Denmark). The normal VOR gain range was taken as 0.8‒1.2 for the horizontal canals and 0.7‒1.2 for the vertical canals.^18^ If the VOR gain was not within the specified range or if there were covert and/or overt catch-up saccades, this was accepted as an abnormal vHIT response and indicated damage related to high-frequency vestibular function.^18^, ^19^

The cervical vestibular evoked myogenic potential (cVEMP) test was conducted to evaluate the function of the saccule (Eclipse EP-25/vEMP, Interacoustics, Taastrup, Denmark). cVEMP responses were obtained after monaural stimulation (500 Hz tone burst acoustic stimulus) in the form of biphasic waveforms, with the first potential being a Positive peak (P1), followed by a Negative peak (N1). In the cVEMP test, the failure to obtain the P1-N1 wave or its low amplitude was accepted as an abnormal response.^13^

The bithermal binaural air caloric test, in which each ear was irrigated with an airflow of 8 L/min at 50 °C and 25 °C for seconds, was performed using the ICS Chart 200 VEG/ENG system (Otometrics, Denmark). The Slow Phase Velocity (SPV) of nystagmus emerging with the horizontal VOR arch was compared between the symptomatic and asymptomatic ears following the stimulation of both labyrinths with air. Canal weakness reflects a reduced vestibular response in the horizontal semicircular canal. SPV being ≥25% in one ear was evaluated as unilateral weakness/canal paresis and ≤12% SPV in both ears was accepted as bilateral weakness, both indicating accepted as a pathological/abnormal response.^20^, ^21^ Canal weakness was calculated based on Jongkee’s formula.^22^

### DHI

DHI is a 25-item instrument developed to determine effect of dizziness/vertigo experienced by adults from their perception. When the item scores are summed, the total score ranges from 100 to 0. High scores show that the dizziness complaint is at a level that impairs daily life activities.^23^ The Turkish version of the questionnaire was used considering the native language of the participants.^24^

### Statistical analysis

Data analysis was employed using SPSS v. 21.0. The descriptive statistics of quantitative variables were shown as mean ± standard deviation, while qualitative variables were presented as counts and percentages. The normality of quantitative variables was evaluated with the Shapiro-Wilk test. The comparison of the PTA values between the symptomatic and asymptomatic ears was performed with the paired-samples *t*-test. The Mann-Whitney *U* test was used for the comparison of non-normally distributed variables between two groups. The correlation between the quantitative variables was evaluated with the Spearman correlation analysis. The Mc-Nemar analysis was conducted to assess differences in dichotomous dependent variables between two-related methods. Sensitivity, specificity, positive predictive and negative predictive values were calculated; p-values less than 0.05 were considered significant.

## Results

Sixteen subjects with unilateral MD (seven females; mean age, 41 ± 9.7 years, nine male; mean age, 44 ± 11.6 years) were included in this study. The mean duration of illness was 38 months (range, 1–96 mounts). The detailed findings are shown in Table 1a, Table 1b.

**Table 1a:** Characteristics of the symptomatic ears of the patients with Meniere’s disease.

PTA, Pure Tone Average; MD, Meniere’s Disease; EH, Endolymphatic Hydrops; PE, Perilymphatic Enhancement; N, Normal; A, Abnormal; cVEMP, Cervical Vestibular Evoked Myogenic Potential; LF, Low Frequencies; HF, High Frequencies; MF, Middle Frequencies; VOR, Vestibulo-Ocular Reflex; vHIT, video Head Impulse Test; R, Right; L, Left; B, Bilateral.

**Table 1b:** Characteristics of the asymptomatic ears of the patients with Meniere’s disease.

PTA, Pure Tone Average; MD, Meniere’s Disease; EH, Endolymphatic Hydrops; PE, Perilymphatic Enhancement; N, Normal; A, Abnormal; cVEMP, Cervical Vestibular Evoked Myogenic Potential; LF, Low Frequencies; HF, High Frequencies; MF, Middle Frequencies; VOR, Vestibulo-Ocular Reflex; vHIT, video Head Impulse Test; R, Right; L, Left; B, Bilateral.

**Figure 1:**
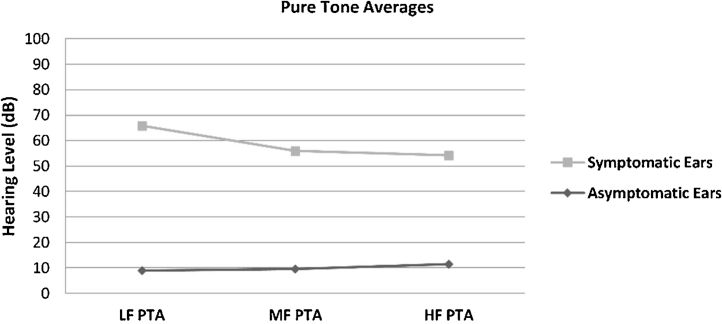
Pure Tone Average (PTA) of conventional hearing thresholds in the symptomatic and asymptomatic ears of the patients with Meniere’s disease. LF PTA: pure tone averages at low frequencies (0, 25, 0.5 and 1 kHz); MF PTA: pure tone averages at middle frequencies (0.5,1 and 2 kHz); HF PTA: pure tone averages at high frequencies (2, 4, 6 and 8 kHz).

The hearing thresholds (both air and bone conduction) statistically significantly differed between the symptomatic and asymptomatic groups (*p* < 0.05) at all frequencies (0.125–8 kHz), with the hearing thresholds of the symptomatic ears being statistically and clinically higher (worse hearing) (*p* < 0.05). No air-bone gap was observed for either side at the audiometric examination. The PTA values are shown in Fig. 1. The PTA values at high-frequency thresholds were significantly related to cEH (*p* < 0.05); i.e., as high-frequency PTA values increased (hearing worsened), the cEH grade increased, while vEH was not significantly correlated with the hearing thresholds (*p* > 0.05).

**Figure 2:**
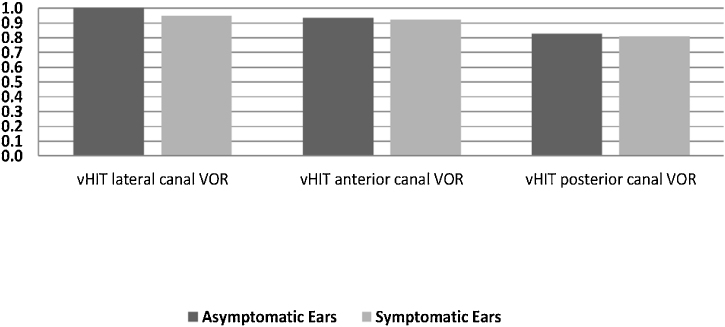
Mean Vestibulo-Ocular Reflex gain (VOR) according to the video Head Impulse Test (vHIT) in the symptomatic and asymptomatic ears.

In the symptomatic group, there were abnormal responses in 14 of the 16 ears (87.5%) in the caloric test, 13 ears (81.3%) in the cVEMP test, and 8 ears (50%) in vHIT. In the asymptomatic group, one ear had an abnormal response in the caloric test and five ears in the cVEMP test, while the vHIT results were completely normal. There was no statistically significant difference between the two sides in terms of the mean VOR gain of any of the SCCs (*p* > 0.05). Fig. 2 presents the VOR gains of the two sides according to the vHIT test.

Isolated cEH was found in 93.7% (15/16) of the symptomatic ears and 68.8% (11/16) of the asymptomatic ears. Isolated vEH was detected at a rate of 87.5% (n = 14) in the symptomatic group and 37.6% (n = 6) in the asymptomatic ears. When considered in general, EH (cEH and/or vEH) was observed in 93.7% of the symptomatic ears and 68.7% of the asymptomatic ears.

In the symptomatic group, vestibular PE (vPE) was detected in 18.7% (n = 3) of the ears and cochlear PE (cPE) in 18.7% (n = 3). When considered in general, PE (cPE and/or vPE) was observed in 37.5% of the symptomatic ears (6/16). In addition, PE was present in six of the asymptomatic ears (cPE: 5 and vPE: 1). PE was present in six of the 15 symptomatic ears which also had EH. Only one symptomatic ear had normal values on MRI (no proof of EH or PE was present); however, the audio-vestibular test battery of this ear showed pathological findings. Data on the sensitivity, specificity, positive predictive and negative predictive values of the methods and parameters used in the study are shown in Table 2.

**Table 2:** Sensitivity, specificity and Positive Predictive Values (PPV) and Negative Predictive Values (NPV) of the methods and parameters used in the study.

(%)	Caloric test	vHIT test	cVEMP test	Vestibular test battery (caloric test + vHIT + cVEMP)	EH (MRI)	PE (MRI)	EH + PE (MRI)
Sensitivity	87.5	50	81.3	100	93.8	37.5	93.8
Specificity	93.8	68.8	68.8	50	31.3	62.5	81.3
Total	181.3	118.8	150.1	150	125.1	100	175.1
PPV	93.3	61.5	72. 2	66.7	57.7	50	83.3
NPV	88.2	57.9	78. 6	100	83.3	50	92.9

EH, Endolymphatic Hydrops; PE, Perilymphatic Enhancement; cVEMP, cervical Vestibular Evoked Myogenic Potential; vHIT, video Head Impulse Test; MRI, Magnetic Resonance Imaging.

**Figure 3:**
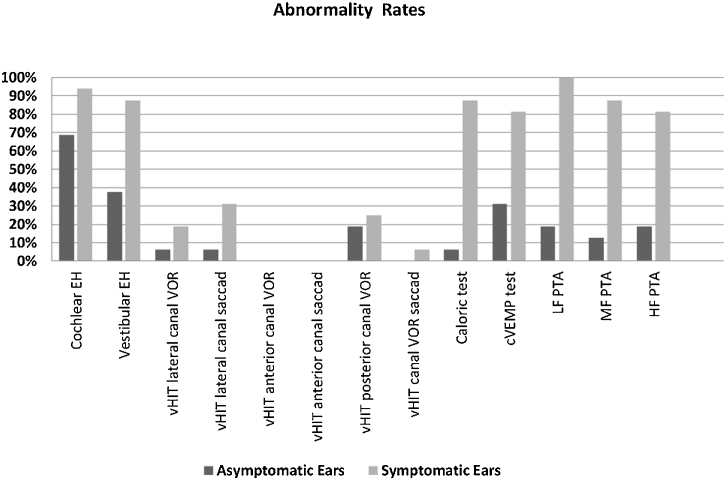
Abnormality rates of the parameters evaluated in the symptomatic and asymptomatic ears of the patients with Meniere’s disease. (EH, Endolymphatic Hydrops; vHIT, video Head Impulse Test; VOR, Vestibulo-Ocular Reflex gain; cVEMP, Cervical Vestibular Evoked Myogenic Potential; PTA, Pure Tone Average; LF, Low Frequencies; HF, High Frequencies; MF, Middle Frequencies).

The rates of vEH, canal paresis, abnormal cVEMP response, and abnormal PTA in the symptomatic ears were statistically significantly higher compared to the asymptomatic ears (*p* < 0.05) (Fig. 3). There was no significant relationship between the vestibular test results and MRI findings (*p* > 0.05). However, a statistically significant relationship was found between all the PTA values and DHI scores, particularly at low frequencies (*p* < 0.05). As the PTA level increased (hearing worsened), the DHI score also increased. There was no relationship between age, MD duration, EH, vestibular test results, and DHI score (*p* > 0.05).

## Discussion

In the current study, while the rate of isolated cEH was similar in the symptomatic and asymptomatic ears, that of isolated vEH was higher on the symptomatic side (Fig. 3). Recent studies show that vEH is more specific for MD than Ceh,^10^, ^25^, ^26^ which is also supported by our data. On the other hand, while the rate of EH (cEH and/or vEH) of the symptomatic ears on MR imaging was similar to previous some studies,^10^, ^16^ the rate of EH on the asymptomatic ears was found to be inconsistent with the literature^16^

The rate of increased PE was very low in our symptomatic group (37.5%). When we applied the algorithm suggested by Bernaerts et al. for the PE and MD relationship,^2^ the PE rate reached only 56.2%. In either case, the evaluation of PE alone was not sufficient for the diagnosis of MD. This shows that increased PE is not an important marker of definite MD. On the other hand, adding PE to the EH evaluation in MRI increased specificity from 0.31 to 0.81 and increased positive predictive value from 0.57 to 0.83. The combination of the EH and PE grading systems makes IV Gd-enhanced inner ear MRI a more reliable technique in MD.

The increase in the hearing thresholds of patients with MD is proof of the progression of the disease.^1^ In our study, the mean disease duration was 38 months, and the hearing thresholds of the symptomatic ears were significantly higher at all frequencies (250‒8000 Hz). The average hearing thresholds at 2, 4, 6 and 8 kHz, obtained by eliminating the risk of age-related hearing loss (presbycusis) as much as possible (mean age, 42.7 years), was significantly correlated with the cEH degree. As the cEH degree increased, the hearing thresholds also increased. Furthermore, we observed a significant relationship between the DHI score and the all-PTA values of the patients with MD, especially the low-frequency hearing thresholds. As the PTA level increased (hearing worsened), the DHI score increased. As a result, as the cEH grade increased, the hearing thresholds and DHI score increased. On the other hand, a similar correlation was not observed between vEH and hearing thresholds.^3^, ^27^, ^28^

In this study, the caloric test evaluating horizontal SSC function revealed an abnormal response in 87.5% of the symptomatic ears, while this rate was 31.3% in the horizontal canal in vHIT. Nevertheless, canal paresis was present in all 11 ears with normal vHIT findings. Previous reports in MD also showed significant differences in the results of vHIT and the caloric test,^3^, ^29^, ^30^ which is in line with our findings. Although both methods cause the deflection of the horizontal SSC cupula, they provide stimulation at different frequencies; low frequency in the caloric test (approximately 0.006 Hz) and high frequency in vHIT (approximately 2.5 Hz).^29^ Therefore, the varying test results may be related to the stimulus applied at different frequencies. We also failed to obtain significant abnormality in the vHIT responses of the anterior and posterior canals in the symptomatic ears. vHIT alone was not considered to be reliable in the diagnosis of MD. On the other hand, studies conducted with larger samples did not report a significant correlation between canal paresis and EH was obtained,^28^, ^31^, ^32^, ^33^ which is in agreement with our data. In studies evaluating patients with MD, abnormal responses in the cVEMP test vary in an extensive range.^34^, ^35^ For example, De Waele et al. reported abnormal results in 54% of patients,^36^ while Seo et al. detected them at a rate of 96%.^31^ In the current study, there was an abnormal response in 81.3% (13/16) of the symptomatic ears. In our study, the MD durations of the three patients with normal responses were reported as one month, 12 months, and 36 months, respectively.^37^, ^38^ In MD, the cVEMP response may decrease or may not be obtained in cases with a longer duration of the disease. Our data confirm that saccular function is significantly affected in MD. The relationship between cVEMP and MRI seems variable and warrants further research.^3^, ^31^ The lack of a significant relationship between cVEMP and vEH may be related to the small number of patients in our sample. In the literature examining MRI and audio-vestibular tests, cEH is mainly associated with PTA, similar to our study. We did not find a significant correlation between the vestibular test results (vHIT, cVEMP, and caloric test) and MRI findings (EH and PE).

The sensitivity of our vestibular test battery (vHIT, cVEMP, and caloric test) was 100%, and its specificity was 50%. The sensitivity and specificity values of IV Gd-enhanced inner ear MRI (EH and PE together) were determined as 93.8% and 81.3%, respectively. We did not find a similar study investigating the same vestibular test battery; however, our MRI (EH + PE) results are similar to those reported by Steekelenburg et al. (sensitivity, 86%; specificity, 97%).^10^ The high rate of EH we detected in the asymptomatic ears and the presence of EH also being reported in different otological diseases other than MD.^39^, ^40^ suggest that EH is a histological marker of MD rather than being directly responsible for the symptoms of the disease.^14^ The invasiveness, high cost and contraindications (cochlear implant use, kidney dysfunction, allergy, etc.) of IV Gd-enhanced inner ear MRI limit the applicability of this technique. In addition, since the severity of symptoms varies between attacks and according to the time of attacks in MD,^1^ the ideal time for MRI with contrast administration is when an MD attack takes place. However, patients mostly present to the emergency department during an attack, and this MRI technique is not available in all centers; therefore, auditory and vestibular tests consisting of pure tone audiometry, caloric test, and cVEMP maintain their popularity.

The main limitation of the current study is that the contralateral clinically normal ears of the patients with definite MD were used as a control group. Since IV Gd-enhanced MRI is considered to have no diagnostic benefit, it was not possible to include healthy control subjects in the study due to ethical considerations. Another limitation concerns the small number of participants. In future studies, the evaluation of large patient groups and gender comparison may provide further valuable data. The final limitation is that all assessments were made during the inactive period of the disease.

## Conclusion

IV Gd-enhanced inner ear MRI (EH and PE together) had higher sensitivity and specificity than the vestibular test battery (cVEMP, vHIT, and caloric test). In the presence of suspected MD, the clinical history, hearing tests, and IV Gd-enhanced inner ear MRI are sufficient for diagnosis. MRI should be first choice, if this technique is not possible, clinical history, hearing tests and vestibular tests (caloric test and cVEMP, not vHIT) can provide reliable results when evaluated together.

## Level of evidence

1 How common is the problem? Level 1
2 Is this diagnostic or monitoring test accurate? (Diagnosis): Level 2
3 What will happen if we do not add a therapy? (Prognosis) Level 5
4 Does this intervention help? (Treatment Benefits) Level 1
5 What are the COMMON harms? (Treatment Harms) Level 1
6 What are the RARE harms? (Treatment Harms) Level 1
7 Is this (early detection) test worthwhile? (Screening) Level 2

## Conflicts of interest

The authors declare no conflicts of interest.
